# Folate Transport and One-Carbon Metabolism in Targeted Therapies of Epithelial Ovarian Cancer

**DOI:** 10.3390/cancers14010191

**Published:** 2021-12-31

**Authors:** Adrianne Wallace-Povirk, Zhanjun Hou, Md. Junayed Nayeen, Aleem Gangjee, Larry H. Matherly

**Affiliations:** 1 Molecular Therapeutics Program, Barbara Ann Karmanos Cancer Institute, 421 East Canfield Street, Detroit, MI 48201, USA; awallace@fredhutch.org (A.W.-P.); houz@karmanos.org (Z.H.); 2 Department of Oncology, Wayne State University School of Medicine, Detroit, MI 48201, USA; 3 Division of Medicinal Chemistry, Graduate School of Pharmaceutical Sciences, Duquesne University, Pittsburgh, PA 15282, USA; nayeenm@duq.edu (M.J.N.); gangjee@duq.edu (A.G.); 4 Department of Pharmacology, Wayne State University School of Medicine, Detroit, MI 48201, USA

**Keywords:** epithelial ovarian cancer, folate, folate receptor, folate transport, one-carbon metabolism, proton-coupled folate transporter, tumor microenvironment

## Abstract

**Simple Summary:**

New therapies are urgently needed for ovarian cancer, the most lethal malignancy in women. To identify new approaches for targeting ovarian cancer, metabolic vulnerabilities must be discovered and strategies for the selective delivery of therapeutic agents must be established. New approaches that are tumor-selective and that facilitate the internalization of novel drugs or provide targets for therapy are being developed for treating ovarian cancer involving folate receptors and the proton-coupled folate transporter. New drugs are being discovered that target key metabolic processes in tumors and neighboring immune cells which contribute to tumor progression. In this review, we describe the remarkable advances in this rapidly evolving area and their extraordinary potential to improve the lives of women diagnosed with this devastating disease.

**Abstract:**

New therapies are urgently needed for epithelial ovarian cancer (EOC), the most lethal gynecologic malignancy. To identify new approaches for targeting EOC, metabolic vulnerabilities must be discovered and strategies for the selective delivery of therapeutic agents must be established. Folate receptor (FR) α and the proton-coupled folate transporter (PCFT) are expressed in the majority of EOCs. FRβ is expressed on tumor-associated macrophages, a major infiltrating immune population in EOC. One-carbon (C1) metabolism is partitioned between the cytosol and mitochondria and is important for the synthesis of nucleotides, amino acids, glutathione, and other critical metabolites. Novel inhibitors are being developed with the potential for therapeutic targeting of tumors via FRs and the PCFT, as well as for inhibiting C1 metabolism. In this review, we summarize these exciting new developments in targeted therapies for both tumors and the tumor microenvironment in EOC.

## 1. Introduction

Epithelial ovarian cancer (EOC) is the leading cause of death in women diagnosed with gynecological cancers in the USA and accounts for ~90% of ovarian cancers [1]. The high mortality for patients diagnosed with EOC reflects the development of chemoresistance to standard cytotoxic chemotherapy in late-stage disease [2]. Clearly, there is an urgent need for new therapeutic strategies that provide longer disease-free intervals and improve overall survival, particularly for patients with platinum-resistant EOC who have limited treatment options.

In recent years, attention has focused on targeted therapies for EOC. While much attention has involved poly(adenosine diphosphate-ribose) polymerase (PARP) inhibitors in patients with *BRCA* mutations and homologous recombination deficiency [3,4], of particular interest is the targeting of EOC through the folate receptor (FR) proteins [5,6,7] and the proton-coupled folate transporter (PCFT) [8,9,10]. FRα is expressed in ~85% of EOCs [6,11,12,13], whereas the PCFT is expressed constitutively in EOCs [14]. Further, as the tumor microenvironment (TME) has emerged as an important determinant of disease progression for EOC, it is notable that tumor-associated macrophages (TAMs) express FRβ, thus providing new opportunities to suppress ovarian tumor progression by inhibiting TAMs with FR-targeted therapies [15,16,17,18].

One-carbon (C1) metabolism has remained an important therapeutic target for many cancers since the introduction of methotrexate over 60 years ago and, more recently, pemetrexed [19,20]. This reflects the central role of C1 metabolism in the synthesis of thymidylate, purines, serine, and methionine as well as in supporting biological methylation reactions from S-adenosylmethionine (SAM) [21,22,23,24,25].

With the recognition that C1 metabolism is compartmentalized between the cytosol and mitochondria, recent attention has focused on the therapeutic potential for targeting mitochondrial C1 metabolism in cancer, reflecting its critical role as a source of C1 units, glycine, reducing equivalents (e.g., NAD(P)H), and ATP [21,22,25]. However, the value of targeting C1 metabolism in EOC has yet to be realized, although there is increasing interest in developing targeted antifolates with FR and/or PCFT selectivity and that inhibit essential metabolic pathways or processes with therapeutic impact [8,10,19,21,26,27].

In this review, we provide an overview of C1 metabolism, including the recent major discoveries in this area. We discuss the potential for exploiting the vulnerabilities of EOC in C1 metabolism with novel antifolates and the selective uptake of targeted therapeutics by FRs and the PCFT in the context of current therapies and the unique biology of EOC.

## 2. Folate Homeostasis, Folate Transport, and One-Carbon Metabolism

### 2.1. Folate Homeostasis and Transport

**Figure 1:**
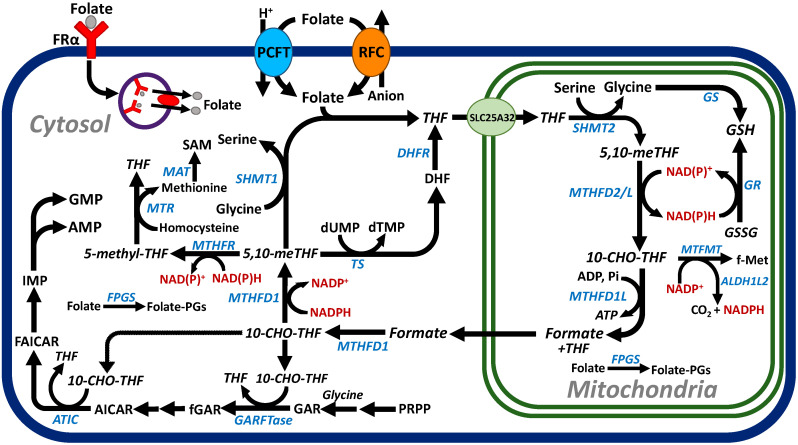
Folate transport and C1 metabolism. A schematic is shown depicting cellular uptake by facilitative transport via RFC or PCFT or by endocytosis via FRα. Intracellular folates are metabolized to polyglutamate conjugates. Folate “monoglutamates” are transported into the mitochondria by SLC25A32. In the mitochondria, serine is catabolized by sequential SHMT2, MTHFD2/L, and MTHFD1L through which the C1 moiety from serine C3 is incorporated into formate, thus providing C1 units for cellular biosynthesis in the cytosol. Abbreviations are as follows: 10-CHO-THF, 10-formyl tetrahydrofolate; 5,10-me-THF, 5,10-methylene tetrahydrofolate; AICAR, 5-aminoimidazole-4-carboxamide; ALDH1L2, aldehyde dehydrogenase 1 family member L2; ATIC, 5-aminoimidazole-4-carboxamide ribonucleotide formyltransferase; DHF, dihydrofolate; DHFR, dihydrofolate reductase; FAICAR, formyl 5-aminoimidazole-4-carboxamide ribonucleotide; fGAR, formyl glycinamide ribonucleotide; FPGS, folylpoly-γ-glutamate synthetase; GAR, glycinamide ribonucleotide; GARFTase, glycinamide ribonucleotide formyltransferase; GR, glutathione reductase; GS, glutathione synthetase; GSH, glutathione; MTFMT, methionyl tRNA formyltransferase; MTHFD1, methylenetetrahydrofolate dehydrogenase 1; MTHFD2(L), methylene tetrahydrofolate dehydrogenase 2(-like); MTHFR, methylenetetrahydrofolate reductase; MTR, methionine synthase; PCFT, proton-coupled folate transporter; PGs, polyglutamates; PRPP, phosphoribosyl pyrophosphate; RFC, reduced folate carrier; SAM, S-adenosylmethionine; SHMT1/2, serine hydroxymethyltransferase 1/2; THF, tetrahydrofolate; and TS, thymidylate synthase.

Folates are members of the vitamin B9 family and are composed of a bicyclic pteridine ring which in cells can be reversibly reduced by dihydrofolate reductase with one (dihydrofolate) or two (tetrahydrofolate (THF)) reducing equivalents, a *p*-aminobenzoate, and an *L*-glutamate. Folates are hydrophilic anionic molecules and have limited capacities to diffuse across cell membranes. Extracellular folates use PCFT (SLC46A1) and RFC (SLC19A1), which are facilitive transporters, as well as FRs, for cellular uptake [6,9,28,29] (Figure 1). Each of these transport systems plays a unique role in the maintenance of C1 homeostasis.

The RFC (SLC19A1) is a folate-organic anion antiporter with optimal activity at pH 7.4 [9], whereas the PCFT (SLC46A1) is a folate-proton symporter that is optimally active at an acidic pH (pH 5.5) [30]. Both RFC and PCFT are members of the major facilitator superfamily of transport proteins [31].

RFC is ubiquitously expressed in tissues and tumors and is the major tissue transporter of folate cofactors (e.g., 5-methyl THF) from the systemic circulation [9,28,29]. RFC is highly expressed in the liver and placenta, with substantial levels in the kidney, lung, bone marrow, intestine, and central nervous system [32]. In mouse tissues, RFC is expressed at the basolateral membrane of renal tubule epithelia, the apical surface of the choroid plexus, hepatocyte membranes, and the apical membrane of cells lining the spinal canal [33]. While RFC is expressed on the apical brush border membrane of the small intestine and colon [33], RFC is not the primary transport system for the absorption of dietary folates in the gastrointestinal (GI) system (see below). Tissue-specific expression of human *SLC19A1* gene is regulated by intricate transcriptional and posttranscriptional controls involving as many as six non-coding exons/promoters [9,32]. This likely reflects the unique tissue requirements for reduced folates and may in part relate to the pathophysiology of folate deficiency. Indeed, low levels of RFC are associated with pathophysiological conditions associated with folate deficiency, ranging from fetal abnormalities to cardiovascular disease, neurologic disorders, and cancer [9]. Further establishing the central role of RFC in folate homeostasis and biology, the loss of the *SLC19A1* gene in the absence of folate supplementation is embryonic lethal, whereas folate supplementation in the absence of RFC is associated with developmental delays and is accompanied by developmental malformations [34,35]. In ovarian cancer, RFC levels did not correlate with disease stage, histologic grade, or histologic subtypes; however, RFC levels correlated with overall and disease-free survival [36].

PCFT was discovered as the major folate intestinal transporter in that it facilitates the absorption of dietary folates at the acidic pH of the upper GI tract [37]. Variants in the human *SLC46A1* gene result in hereditary folate malabsorption due to a loss of PCFT expression or transport activity and are accompanied by severely low levels of systemic and cerebral folates [38]. Although PCFT is also expressed in the choroid plexus, kidney, liver, placenta and spleen [8,29,39], it is not considered to be a major transporter of folates in most normal tissues. Further, even when expressed, PCFT transport activity is very modest in most tissues outside of the upper GI due to a bicarbonate inhibition of transport at a neutral pH [40].

Interestingly, PCFT is highly expressed in a variety of solid tumors, including colorectal adenocarcinoma, malignant pleural mesothelioma, non-small cell lung cancer, and EOC [14,41,42,43]. Conversely, PCFT is expressed at very low levels in leukemias. In at least some cases, low levels of PCFT in human tissues reflect *SLC46A1* promoter hypermethylation, as PCFT can be induced by treatment with 5-aza-2′-deoxycytidine [42,44,45]. Given its substantial transport activity at an acidic pH, including those associated with the low pH conditions of the tumor microenvironment [46], there is growing interest in developing targeted therapeutics for cancer with PCFT transport selectivity over that mediated by RFC [10,19,41].

FRs consist of α, β, γ, and δ isoforms encoded from a multigene family (*FOLR1*, *FOLR2*, *FOLR3*, and *FOLR4*, respectively). FRα, FRβ, and FRδ are tethered to the cell membrane through a glycosylphosphatidylinositol (GPI) linkage, whereas FRγ is not [6,47]. FRα is unique in its tissue specificity in that it is expressed on apical membranes in a small number of healthy epithelial tissues, including those of the female reproductive tract, as well as the kidney, lung, choroid plexus, and placenta [6,12,13,48,49]. FRα serves an important function in embryogenesis [50]. FRβ is expressed in placenta, mature neutrophils, and activated monocytes and macrophages [51,52,53]. FRγ is secreted and is expressed in hematopoietic tissues [54]. FRδ is expressed in the membranes of gametes [47]. (Anti)folate uptake by the membrane-associated FRs involves the high-affinity binding of folate at the cell surface, followed by endocytosis through which the FRs on the cell surface are internalized due to the formation of cytosolic vesicles [55]. These vesicles are acidified to effect dissociation of the bound ligand, which is subsequently released to the cytosol [55]. Compared to facilitated (anti)folate transport by RFC and PCFT, cellular uptake through endocytosis by the GPI-linked FRs is inefficient [6,29].

FRα is expressed on the membrane surface of non-small cell lung cancers, triple negative breast cancers, and kidney, endometrial, and colorectal cancers [6,11,12,13,48,49]. Pancreas, brain, gastric, liver, prostate, and bladder cancers are reported to express FRα in lower levels [6,11,12,13,48,49]. In EOC, FRα is highly expressed and increases with disease stage [12,14,36,56]. FRα in tumors is generally accessible to the circulation. However, in normal (polarized) tissues, with the exception of the placenta, FRα localizes to luminal membranes without exposure to systemic circulation [6,11,13]. These features provide a compelling rationale for developing selective FR-targeted therapeutics for solid tumors. The *FOLR1* gene encoding FRα includes two TATA-less promoters (P1, P4) [57]. In malignant cells, the *FOLR1* P4 promoter appears to predominate. Tissue-specific expression of FRα involves both transcriptional and post-transcriptional mechanisms, including alternate *FOLR1* promoters and mRNA splicing [57], as well as differential translation of mRNAs [58]. In contrast to FRα, FRβ is transcribed from a single *FOLR2* promoter [59]. The *FOLR1* P4 promoter is repressed by estrogen receptor [60] and activated by dexamethasone [61]. In ovarian cancer, FRα may [36] or may not [62] be associated with *FOLR1* gene amplification (chromosome 11q13.3).

### 2.2. C1 Metabolism

C1 metabolism and folates are compartmentalized in the cytosol and the mitochondria of mammalian cells (Figure 1) [21,22,25]. Following internalization, cytosolic folates are transported into the mitochondria by a mitochondrial folate transporter (SLC25A32) [63,64]. Both cytosolic and mitochondrial folates are metabolized to polyglutamate conjugates, catalyzed by alternate isoforms of folylpoly-γ-glutamate synthetase (FPGS) encoded by a single *FPGS* gene [65]. FPGS catalyzes the addition of up to eight additional glutamate residues to the γ-carboxyl of the terminal glutamate of the parent folate molecule [66]. Once polyglutamylated, cytosolic folates are preferentially retained in cells [66] and do not exchange with the mitochondrial folate polyglutamyl forms [65]. For most C1 transfer reactions, polyglutamyl folate forms are the preferred substrates over the non-polyglutamyl or monoglutamate folate forms [66].

Serine is synthesized from glucose via the glycolytic intermediate 3-phosphoglycerate. Phosphoglycerate dehydrogenase reduces 3-phosphoglycerate to 3-phosphohydroxypyruvate, which is then converted to serine via transamination and phosphoserine hydrolysis reactions. Serine serves as the principal source of C1 units for cellular biosynthesis. Following its transport into the mitochondria [67], serine is metabolized sequentially by serine hydroxymethyl transferase (SHMT) 2 to glycine and 5,10-methylene THF (Figure 1). The 5,10-methylene THF is oxidized in an NAD(P)^+^ dependent reaction by 5,10-methylene THF dehydrogenase (MTHFD) −2 or −2L to 10-formyl THF. The 10-formyl THF is hydrolyzed by MTHFDL1 to THF and formate, which passes to the cytosol for utilization in C1 biosynthetic reactions (Figure 1) [21,22,68,69,70]. Serine catabolism in the mitochondria serves as the principal source of C1 units and glycine for cellular biosynthesis, including the de novo synthesis of purine nucleotides and thymidylate in the cytosol [21,22,68,69,70]. Further, mitochondrial C1 metabolism is an important source of NAD(P)H and glycine for the synthesis of glutathione as well as ATP (Figure 1) [21,22,68,69,70]. Mitochondrial NADPH is also generated by the catabolism of 10-formyl-THF to CO_2_ and THF through aldehyde dehydrogenase 1 family member L2 (ALDH1L2) [71].

In the cytosol, 10-formyl THF is resynthesized from THF and formate by the 10-formyl THF synthetase activity of the trifunctional enzyme MTHFD1 (Figure 1). The MTHFD1 cyclohydrolase and dehydrogenase activities further convert the 10-formyl THF to 5,10-methylene THF and the oxidation of NADPH to NADP^+^. For de novo purine biosynthesis, 10-formyl THF is required, which involves 10 reactions from phosphoribosyl pyrophosphate (PRPP) to IMP [72] (Figure 1). Glycine is incorporated into glycinamide ribonucleotide (GAR) by GAR synthetase. C1 units from 10-formyl THF are used in reactions catalyzed by glycinamide ribonucleotide formyl transferase (GARFTase) and 5-aminoimidazole-4-carboxamide ribonucleotide (AICAR) formyl transferase (ATIC). Thus, both the glycine and formate resulting from mitochondrial C1 metabolism are incorporated into the purine ring. To facilitate an efficient flux of pathway intermediates, de novo purine biosynthetic enzymes are assembled into a “purinosome” [73], which mediates passage of the key intermediates, including the THF cofactors between the sequential steps from PRPP to AMP and GMP. Purinosomes are reported to co-localize with mitochondria to further maximize the metabolic efficiency of purine biosynthesis [73].

SHMT1 is the cytosolic homolog of SHMT2 in mitochondria and catalyzes the reverse reaction, whereby glycine is converted to serine with a C1 unit from 5,10-methylene THF, forming THF (Figure 1). Thymidylate synthase converts dUMP to thymidylate, with 5,10-methylene THF providing a C1 unit and reducing equivalent, generating dihydrofolate. Dihydrofolate is reduced back to active THF by dihydrofolate reductase. 5,10-Methylene THF can also be reduced to 5-methyl THF in a NAD(P)H-dependent reaction catalyzed by 5,10-methylene THF reductase (MTHFR) (Figure 1). 5-Methyl THF provides C1 units for the vitamin B12-dependent conversion of homocysteine to methionine catalyzed by methionine synthase (MTR). Methionine is further metabolized to SAM by methionine adenosyltransferase (MAT), required for the methylation of DNA, phospholipids, and proteins [74] (Figure 1). DNA methylation mediates the silencing of transcription and aberrant gene promoter methylation. The role of DNA methylation in the progression and chemoresistance in high-grade serous ovarian cancer at both the genome-wide and individual gene level has been recently reviewed [75].

Mitochondrial SHMT2 and serine provide C1 units for cellular anabolism in the cytosol. The opposing directionality of the C1 flux in each compartment (i.e., serine-to-formate in the mitochondria; formate-to-serine in the cytosol) reflects differences in the electrochemical potential between the mitochondrial NAD(P)H [high NAD(P)^+^/NAD(P)H] and cytosolic NADPH [low NAD(P)^+^/NAD(P)H] [25]. Without this, the cytosolic NAD^+^ pools would soon be depleted, resulting in decreased glycolysis accompanying serine oxidation and the generation of NADH [22]. Interestingly, mitochondrial C1 also stabilizes the THF from degradation in the cytosol by providing formate for the synthesis of 10-formyl-THF by MTHFD1 [76].

Notably, serine catabolism is elevated in cancer, and the genes encoding MTHFD2 and SHMT2 are among the top five differentially expressed genes in tumors and normal tissues for a wide range of cancers, including EOC [77]. Their high levels of expression in cancer cells may reflect their regulation by MYC as MYC binds to the *SHMT2* and *MTHFD2* gene promoters, along with that for *MTHFD1L* [78,79]. Whereas MTHFD2 is considered a tumor-selective target [80,81], a recent study reported that normal and cancer cells express both MTHFD2 and MTHFD2L [81].

## 3. Therapeutic Challenges of Treating Ovarian Cancer

For 2021, it is estimated that there will be 21,410 new cases of ovarian cancer and that an estimated 13,770 women will die of this disease [82]. Ovarian cancer accounts for 5% of cancer deaths among women, far greater than any other gynecologic cancer [1]. Ovarian cancer is a dismal disease that is frequently diagnosed at a late stage. The 5-year overall survival rate is less than 40% [83]. Women present at an advanced stage due to a lack of early detection strategies and symptoms frequently being confused with benign conditions [84,85]. The World Health Organization classification includes six subtypes of epithelial ovarian tumors including serous, mucinous, endometroid, clear cell, transitional cells, and squamous carcinoma [86]. Approximately 10% of all ovarian cancers are non-epithelial (i.e., germ cell tumors, sex cord origin, small cell carcinomas, and sarcomas) [87]. Of the EOC subtypes, 70% are classified as high-grade serous adenocarcinomas [88].

Although ovarian cancer is a disease of aging with >80% of the cases diagnosed in postmenopausal women over age 50, family history and genetics nonetheless play a role in the risk of disease [86]. Women who have a first-degree relative with ovarian cancer have nearly a two-fold increased risk of disease. However, even when genetic testing is performed after diagnosis, only a small number of cases have identifiable and actionable mutations. In terms of mutation status, 5–15% of ovarian cancers show *BRCA* mutations [89,90]. Germline *BRCA1* and *BRCA2* mutations are present in 14–15% of EOCs [91,92] and up to 22.6% of high-grade serous ovarian cancers [91,92]. Somatic *BRCA1* and *BRCA2* mutations have been reported in 6–7% of high-grade serous EOCs [93]. *BRCA1* promoter hypermethylation occurs in 10–20% of high-grade serous ovarian cancers [94,95]. Other reported mutations include Fanconi anemia genes (*PALB2*, *FANCA*, *FANCI*, *FANCL*, and *FANCC*), RAD genes (*RAD50*, *RAD51*, *RAD51C*, and *RAD54L*) and DNA damage response genes (*ATM*, *ATR*, *CHEK1*, and *CHEK2*) [96]. More than 80% of ovarian cancers have *p53* gene mutations [97,98].

Treatment of ovarian cancer has largely remained unchanged over several decades and generally consists of debulking surgery, followed by six-to-eight cycles of chemotherapy. The principal goal of surgery is to achieve complete cytoreduction of all macroscopic disease, as this is associated with a substantial increase in long-term survival and progression-free survival (PFS) [99,100,101]. The standard therapeutic regimen for ovarian cancer includes a combination of paclitaxel and carboplatin administered intravenously once every three weeks [102]. Three or more cycles of neoadjuvant chemotherapy prior to the debulking surgery and adjuvant chemotherapy is an alternative for some patients [103] and provides an opportunity to test for chemosensitivity a priori to identify patients at higher risk of relapse. Most women initially respond to platinum-based therapies; however, subsequent rounds of therapy generally lead to less efficacy and eventually to chemotherapy resistance [104]. Patients are initially characterized as platinum sensitive if their progression-free interval is more than 6 months, and patients who have disease progression in less than 6 months are considered platinum resistant [104].

In recent years, treatment has been increasingly concentrated in specialized centers with an emphasis on centralizing ovarian cancer care and through the use of multidisciplinary teams [105,106]. Targeted and combination therapies have assumed a greater role in the treatment of ovarian cancer. Combination therapies with a platinum-based agent and gemcitabine and/or bevacizumab are extensively used and show significantly better PFS than platinum monotherapy [107]. An exciting advancement has been the development of PARP inhibitors (e.g., olaparib, rucaparib, and niraparib) which are less toxic than the standard platinum agents for both treatment and maintenance therapies [4,108]. PARP inhibitors interfere with the ability to repair DNA damage and exploit *BRCA1* and −2 germline mutations and deficiencies in the DNA damage response which collectively are believed to occur in up to 50% of high-grade EOCs [4].

## 4. The Role of FRα in the Treatment of EOC

FRα is expressed in ~85% of EOCs [6,11,12,13]. Differences in FRα expression have been reported in all of the different histologic subtypes of ovarian cancer [109]. FRα expression increases with disease stage and can span a wide range within a particular subtype [12,14,56]. These findings have prompted interest in using FRα for therapeutic targeting of EOC, particularly for women whose tumors express substantial levels of FRα. Further rationale includes the narrow expression of FRs in non-malignant tissues and the comparatively inefficient uptake of folate substrates compared to facilitative folate transporters, such as the RFC or PCFT [6,9,29]. Different approaches for targeting FRα in EOC patients include monoclonal antibodies, antibody-drug conjugates, folate drug conjugates, and targeted antifolates [110].

Farletuzumab is a humanized IgG1 antibody that targets FRα. Farletuzumab promotes cell death via antibody-dependent cytotoxicity and complement-dependent cytotoxicity, autophagy, and inhibition of Lyn kinase signaling [111,112,113,114]. Farletuzumab was evaluated in a phase I clinical trial with manageable toxicities [115]. A phase II clinical trial with ovarian cancer patients who received farletuzumab, carboplatin, and a taxane, followed by maintenance therapy with farletuzumab, showed a promising overall response rate [116]. A phase III randomized, placebo-controlled trial assessed the efficacy and safety of farletuzumab combined with weekly paclitaxel in platinum-resistant recurrent or refractory EOC (MORAb-003-003; NCT00738699) [117] (Table 1). This study was terminated early after recruiting 417 patients because it did not meet the pre-specified criteria for continuation. A phase III randomized, double-blind, placebo-controlled trial (MORAb-003-004; NCT00849667) compared the efficacy and safety of carboplatin and a taxane with and without weekly farletuzumab in patients with platinum-sensitive ovarian cancer in their first relapse [116,118] (Table 1). Altogether, 1100 women were randomized to three arms (1.25 mg/kg farletuzumab plus combination therapy, 2.5 mg/kg farletuzumab plus combination therapy, or placebo plus combination therapy). No significant differences in PFS among the treatment arms were observed. While the primary PFS endpoint was not achieved, there was a trend toward improved PFS in some patient subsets.

Mirvetuximab soravtansine (IMGN853) is an antibody-drug conjugate to FRα, developed by Immunogen, which was “fast-tracked” toward clinical trials with the objective of determining the clinical efficacy against platinum-resistant ovarian cancers. Mirvetiximab soravtansine consists of a humanized anti-FRα antibody conjugated to a cytotoxic payload of maytansinoid DM4 [119]. An initial study showed that mirvetuximab soravtansine was well tolerated as a single agent, and an expansion phase study in patients with platinum-resistant FRα-overexpressing ovarian cancer afforded promising results (overall response rate of 26%) [120]. In a randomized, open-label, phase III study (FORWARD I), which compared mirvetuximab soravtansine and chemotherapy in 366 patients with platinum-resistant EOC, mirvetuximab soravtansine did not result in a significant improvement in PFS compared with chemotherapy (Table 1). Secondary endpoints consistently favored mirvetuximab soravtansine, particularly in patients with high FRα expression. Mirvetuximab soravtansine showed a more manageable safety profile than chemotherapy [121]. Another Phase III clinical trial (IMGN853-0416; NCT04209855) is recruiting patients and is designed to compare the efficacy and safety of mirvetuximab soravtansine versus investigator’s choice chemotherapy in patients with platinum-resistant high-grade EOC, primary peritoneal cancer, or fallopian tube cancer, whose tumors express a high level of FRα.

Another approach for targeting FRα was pioneered by Endocyte in collaboration with Purdue University. This concept involved a conjugate delivery system, including a folic acid-targeting moiety, which binds to exposed FRs on the surface of FR-expressing tumors, permitting internalization by endocytosis [5]. The targeting entity is conjugated to a cytotoxic “warhead”, separated by a hydrophilic cleavable linker, permitting the release of the cytotoxic moiety. Perhaps the most successful conjugate of this series was EC145 (Vintafolide), which included a folic acid-targeting moiety conjugated to desacetylvinblastine monohydrazide (DAVLB), a derivative of the microtubule inhibitor vinblastine [5]. This agent is specific for FR-expressing tumors such that, following its internalization and DAVLB release from the vesicle, EC145 inhibits dividing cancer cells by disrupting mitotic spindle formation. The preclinical data with EC145 were promising, leading to near cures in a high FRα-expressing KB tumor model in immune-compromised mice with limited toxicity. Based on the preclinical data, EC145 advanced to clinical trials [122]. A phase I clinical trial showed an acceptable toxicity profile [123]. A randomized phase II study in women with platinum-resistant ovarian cancer treated with EC145 and pegylated liposomal doxorubicin versus pegylated liposomal doxorubicin alone showed a benefit in PFS for the combined treatment [124]. However, EC145 did not progress past phase III as there was a lack of improvement in PFS for women with predominantly FRα-positive platinum-resistant EOC (Table 1) [125].

**Table 1:** Phase III clinical trial involving FRα-targeted therapies.

Farlatuzumab			
Title	Patients	Results	Reference
Randomized, double-blind, placebo-controlled, phase III study to assess the efficacy and safety of weekly MORAb-003 in combination with carboplatin and taxane in subjects with platinum-sensitive ovarian cancer in first relapse (MORAb-003-004; NCT00849667)	Platinum-sensitive EOC (1100 patients) in first relapse	No significant differences in PFS among the treatment arms were observed. The primary end point of PFS was not met.	[116,118]
Phase III randomized clinical trial of weekly paclitaxel with or without farletuzumab (MORAb-003-003; NCT00738699)	Platinum-resistant ovarian cancer (417 patients)	Study was terminated due to failure to meet pre-specified criteria.	[117]
Vintafolide (EC145)			
Study for women with platinum resistant ovarian cancer evaluating EC145 in combination with Doxil^®^ (PROCEED) (EC-FV-06; NCT01170650)	FRα-positive platinum-resistant ovarian cancer (640 patients)	Trial was terminated owing to failure to meet pre-specified PFS criteria.	[125]
Mirvetuximab soravtansine			
Phase III RCT (FORWARD I) evaluating chemotherapy (paclitaxel, pegylated liposomal doxorubicin, or topotecan) vs. mirvetuximab soravtansine (IMGN853-0403; NCT02631876)	FRα-positive platinum-resistant ovarian cancer (366 patients)	Mirvetuximab soravtansine did not result in a significant improvement in PFS compared with chemotherapy.	[121]

**Figure 2:**
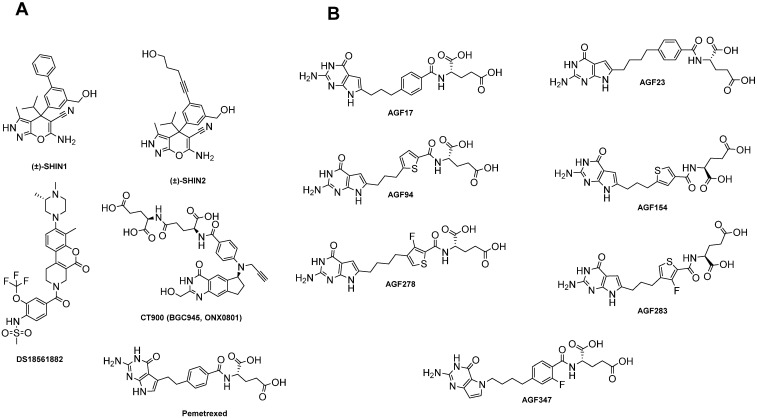
Structures of inhibitors of C1 metabolism. Panel (**A**): structures are shown for the dual SHMT1 and SHMT2 inhibitors SHIN1 and SHIN2 [150,151], the MTHFD2 inhibitor DS18561882 [152], as well as pemetrexed [137] and CT900 (BGC945, ONX0801) [26] (both principally thymidylate synthase inhibitors). Panel (**B**): structures are shown for pyrrolo[2,3-*d*]pyrimidine GARFTase inhibitors, AGF17 [145], AGF23 [145], AGF94 [147], AGF154 [148], AGF278 [149], and AGF283 [149], as well as the pyrrolo[3,2-*d*]pyrimidine antifolate AGF347 [153], which acts as a multitargeted inhibitor of SHMT2 in the mitochondria and of SHMT1, GARFTase, and ATIC in the cytosol.

CT900 (BGC945, ONX0801) is a cyclopenta[g]quinazoline-based antifolate that targets TS (Figure 2, Table 2). CT900 is transported solely by FR rather than the RFC, thus protecting bone marrow and intestinal cells [26]. Preclinical in vivo studies using KB tumor xenografts showed that the terminal half-life of CT900 was longer in tumors than in normal tissues, such as liver and kidney [26]. In 2017, CT900 completed a successful phase I clinical trial with high-grade serous ovarian cancers overexpressing FRα [126]. A retrospective data analysis of an expansion cohort from the trial suggested that 50% of patients with FRα-overexpressing high-grade serous ovarian cancer had a partial response [126].

Cancer imaging through the FRα is complementary to FRα-targeted therapies for EOC. Approaches include FRα-targeted contrast-enhanced magnetic resonance imaging (MRI) [127,128] and radiolabeled folate derivatives [124,129,130]. The latter have been tested in clinical trials, where ^111^In-diethylenetriaminepentaacetic acid-folate was used for whole-body single-photon emission computed tomography in women with endometrial or ovarian cancer [129]. A folate conjugate (etarfolatide) conjugated with ^99^mTc was investigated as a predictive biomarker in clinical trials [124,130], including a phase II clinical trial with vintafolide in women with recurrent platinum-resistant ovarian cancer [124]. The antibody-based agent ^89^Zr-DFO-M9346A was used to measure FRα in murine ovarian cancer xenografts treated with mirvetuximab soravtansine [131].

Fluorescent folate probes have been developed for intraoperative visualization and improved resections of FRα-expressing ovarian, breast, and lung cancers [132]. Folate conjugated to fluorescein isothiocyanate (EC17) was tested intraoperatively in women with ovarian cancer [133,134]. In November 2021, the U.S. Food and Drug Administration approved Cytalux (pafolacianine) [135] for intraoperative visualization of FRα-expressing ovarian cancer lesions.

## 5. Targeting EOC via Targeted Antifolates: C1 Metabolism as a Unique Vulnerability for EOC

Pemetrexed (Alimta) has been used both singly and in combination therapy with recurrent platinum-sensitive and resistant EOC and showed a favorable toxicity profile and response rate comparable to other agents used in first-line combination therapy [136]. Pemetrexed inhibits thymidylate synthase with secondary inhibitions of de novo purine biosynthesis at GARFTase and ATIC [137]. Further, as noted above, the thymidylate synthase inhibitor CT900 (ONX0801) is transported into EOCs by FRα [26] and has shown promising clinical activity toward FRα-overexpressing high-grade serous ovarian cancer [126].

Of particular interest are novel investigational drugs that target the potential metabolic vulnerabilities in EOC, such as de novo purine nucleotide biosynthesis. The expression of GARFTase and ATIC in high-grade serous ovarian cancer from patients (*n* = 39) is dramatically increased (5.2- and 4.4-, respectively) over the levels in normal ovaries (*n* = 8) (A. Wallace-Povirk, L.H. Matherly, unpublished), suggesting the importance of this key anabolic pathway to the malignant phenotype. By extension, anti-purine inhibitors are particularly suited for EOC as they are cytotoxic independent of *p53* and *BRCA* mutant status [138,139]. Further, they are tumor-selective, resulting from the loss of purine salvage [140,141], and effect a suppression of mTOR signaling [142,143].

Particularly notable are 6-subsituted pyrrolo[2,3-*d*]pyrimidine antifolates related to pemetrexed (a 5-substituted pyrrolo[2,3-*d*]pyrimidine benzoyl antifolate with a 2-carbon bridge) (Figure 2B; Table 2). Whereas pemetrexed is a good substrate for both the RFC and PCFT, it is poorly transported by FRα [19]. However, the related 6-substituted pyrrolo[2,3-*d*]pyrimidine benzoyl compounds AGF17 and AGF23 (Figure 2B) are transported by both FRα and PCFT with negligible transport by the RFC [144,145]. A 3-carbon bridge length appears to be optimal for PCFT transport [27,144,146,147]. Moreover, PCFT transport is further enhanced by a replacement of the side-chain phenyl ring by a 2′,3′- and 2′,4′-thiophene, as in AGF94 and AGF154, respectively (Figure 2B) [41,147,148]. Side-chain 3′-fluorinated thienoyl analogs of this series (AGF278, AGF283; Figure 2B) were also synthesized and show high levels of FRα- and PCFT-targeted activity [149].

AGF94, AGF154, AGF278, and AGF283 are potent (nanomolar) inhibitors of EOC cell lines (IGROV1, SKOV3, A2780) with a wide (~3000-fold) range of FRα levels and relatively constant PCFT, although the uptake by FRα predominated when both systems were present [14,149]. In IGROV1 cells, FRα knockdown (~95%) had a modest impact on in vitro drug efficacy, establishing transport redundancy for pyrrolo[2,3-*d*]pyrimidine antifolates such as AGF94 by the PCFT and FRα [14,149]. For AGF94, this finding was extended in vivo [14].

As EOCs express FRα over an extraordinarily wide range [14], the notion of targeting the PCFT in EOC is especially appealing. The PCFT is constitutively expressed in EOC [14] and has a modest expression in most normal tissues [8,29,39]. Further, PCFT is active under the acidic pH conditions associated with the tumor microenvironment, although significant PCFT transport is detectable up to pH 7 [154]. Above pH 7, PCFT transport activity is low [30]. Notably, the recent failures of FRα-targeted therapies in clinical trials with ovarian cancer patients (Table 1) reflect, at least in part, the challenge in identifying FRα-positivity, hence the patients most likely to respond to FR-targeted treatments. This suggests opportunities for the PCFT-targeted therapeutics for EOC independent of FRα expression.

**Table 2:** FR and PCFT-targeted antifolates.

Inhibitor	Transporter	Intracellular Target	References
Pemetrexed	PCFT, RFC	Thymidylate synthase, GARFTase, ATIC	[137]
AGF17	FRα, FRβ, PCFT	GARFTase	[144,145]
AGF23	FRα, FRβ, PCFT	GARFTase	[145]
AGF94	FRα, FRβ, PCFT	GARFTase	[14,147]
AGF154	FRα, FRβ, PCFT	GARFTase	[148]
AGF278	FRα, FRβ, PCFT	GARFTase	[149]
AGF283	FRα, FRβ, PCFT	GARFTase	[149]
AGF347	FRα, PCFT, RFC	SHMT1, SHMT2, GARFTase, ATIC	[153,155,156]
CT900	FRα	Thymidylate synthase	[26]
(±) SHIN1	Not determined	SHMT1, SHMT2	[150]
(+) SHIN2	Not determined	SHMT1, SHMT2	[151]
DS18561882	Not determined	MTHFD1, MTHFD2	[152]

With the recognition of the essential role of mitochondrial C1 metabolism (Figure 1) in malignancies including EOC [77], attention has turned toward identifying the inhibitors of this pathway [21]. SHMT2 and MTHFD2 are highly expressed in EOC [77,157], suggesting the therapeutic potential for targeting this pathway in this disease. While MTHFD2 inhibitors have been reported [152,158,159,160], to date the most promising compound is DS18561882 (Figure 2A; Table 2). DS18451882 is a potent (nanomolar) inhibitor of MTHFD2 with a 90-fold selectivity for MTHFD2 over MTHFD1 [152]. Further, DS18561882 showed in vivo efficacy against MDA-MB 231 triple-negative breast cancer xenografts in immune-compromised mice with minimal toxicity [152]. The roles of FRα, RFC, and PCFT in cellular uptake and anti-tumor efficacy of DS18561882 are unclear.

Rabinowitz and his colleagues reported pyrazolopyran inhibitors of SHMT1 and −2 (SHIN1, SHIN2) (Figure 2A; Table 2) that inhibited the proliferation of human tumor cell lines at sub-micromolar concentrations [150,151]. Notably, SHIN2 circumvented the apparent pharmacologic shortcomings of SHIN1 and showed in vivo efficacy toward a PDX T-cell leukemia mouse model [151]. Of particular interest is the 5-substituted pyrrolo[3,2-*d*]pyrimidine antifolate AGF347 [153,155] (Figure 2B; Table 2). AGF347 is a broad-spectrum anti-tumor agent with demonstrated in vitro activity toward colon cancer, lung adenocarcinoma, and pancreatic adenocarcinoma cell lines [153,155], as well as impressive in vivo efficacy toward MIA PaCa-2 pancreatic cancer xenografts [153]. AGF347 is transported by both FRα and PCFT, as well as RFC, and is a potent inhibitor of SHMT2 in the mitochondria and of SHMT1, GARFTase, and ATIC in the cytosol [153,155,156]. AGF347 inhibited the in vitro proliferation of EOC cell lines including cisplatin-resistant SKOV3 EOC, TOV112D, and A2780 EOC cells; the in vivo anti-tumor efficacy was demonstrated with SKOV3 EOC xenografts in SCID mice [156]. For both SHIN1/2 and AGF347, inhibition of both SHMT1 and SHMT2 was essential to their anti-tumor effects, as this prevents metabolic compensation for the loss of SHMT2 activity by reversal of the SHMT1 reaction and the synthesis of glycine and 5,10-methylene THF [150,153,161].

## 6. Targeting the Tumor Microenvironment in Epithelial Ovarian Cancer

### 6.1. The Role of the Dynamic TME in EOC Progression

Ovarian cancer involves a complex intraperitoneal milieu, which includes not only cells within the bulk tumor, but also fibroblasts, endothelial cells, and assorted immune cells [83,162]. Tumor and non-tumor cells secrete a host of bioactive constituents, including growth factors, hormones, phospholipids, and cytokines, that contribute to the TME [83,162]. Thus, the TME in ovarian cancer involves a dynamic interplay between the malignant ascites and the surrounding tissues via receptor-mediated (e.g., autocrine and paracrine) or contact-dependent signaling and epigenetic regulation that enables tumor progression and immune evasion [83].

Fibroblasts synthesize matrix-metalloproteases and other proteins comprising the extracellular matrix, including collagens, fibronectin, and laminin [163]. Cancer-associated fibroblasts (CAFs) promote tumor cell proliferation, invasion, and migration and also promote immune suppression and angiogenesis [164,165,166,167]. Endothelial cells line blood vessels and are associated with angiogenesis in response to angiogenesis activators (e.g., VEGF and PDGF) and inhibitors (e.g., angiopoetin) [168,169]. Immune cells include macrophages, dendritic cells, myeloid-derived suppressor cells (MDSCs), and lymphocytes that impact both tumor progression and suppression, as well as participate in tumorigenesis, metastasis, and angiogenesis [170,171].

The following section describes strategies for leveraging advances in the biology of FRs and C1 metabolism that target the TME in ovarian cancer with a particular focus on T lymphocytes and TAMs.

### 6.2. Targeting the TME in EOC

The ascites contains a host of infiltrating immune cells, including T lymphocytes and TAMs, although TAMs are considered the principal immune cellular component that results in an immunosuppressive environment in EOC [172,173]. Further, TAMs contribute to metastasis and angiogenesis by releasing pro-angiogenic factors, such as vascular endothelial growth factor and matrix metalloproteinase [162,174].

Initial optimism for the use of immune checkpoint inhibitors with EOC was tempered by disappointing results with these agents in clinical trials as single agents or combined with standard chemotherapy [175]. Nonetheless, the lymphoid compartment remains an intriguing target for novel TME therapies in EOC [83]. Patients with increased levels of CD3+ tumor-infiltrating lymphocytes (TILs) showed delayed recurrence of disease; when greater TIL infiltration was accompanied by increased levels of interferon γ, the EOC patients experienced longer overall survivals [83].

The high levels of FRα in ovarian cancer suggested opportunities for a targeted immunotherapy of EOC employing CAR T. Engineered CAR T cells containing an FRα-specific epitope coupled to the T cell receptor chain CD3ζ induced regression in preclinical models [176]. Based on these results, a phase I clinical trial (NCT03585764) is recruiting patients with ovarian or fallopian tube cancers or primary peritoneal cancers. A recent study described a novel tandem CAR encoding an anti-FRα scFv, an anti-mesothelin scFv, and two peptide sequences of IL-12 to improve the efficacy, infiltration, persistence, and proliferation of CAR T cells in ovarian cancer [177].

There has been increasing interest in targeting TAMs as a cancer therapy, as inhibiting TAMs could, in principle, suppress tumor progression [162]. A screen to discover differentially expressed genes in the presence of macrophages skewed toward an M1-like or M2-like state, revealed that *FOLR2*, which encodes FRβ was differentially expressed in M2-like human macrophages. FRβ was expressed on IL-10-producing M2-like macrophages (CD163+, CD68+, CD14+ IL-10), corresponding to the anti-inflammatory/pro-tumor TAM subtype [16].

As FRβ expression on TAMs was induced by malignant ascites and conditioned media from fibroblasts, it was suggested that the presence of FRβ on the surface of macrophages could offer an opportunity for depleting TAMs with cytotoxic folate-conjugates or antifolates as a component of therapy [16]. In both lung adenocarcinoma and squamous cell carcinoma, FRβ showed an increased expression that was predictive of a worse outcome and prognosis [18]. A BIM (BCL-2-interacting mediator of cell death) plasmid encapsulated in a folate “lipoplex” was used to target the TME of lung cancer. This therapeutic was selectively delivered to FRβ-expressing TAMs, resulting in their substantial depletion in the TME without significant off-target toxicities. Importantly, there were significantly fewer tumor nodules in the lungs of the treated mice compared to the control mice [18]. In an experimental C6 rat glioma model, targeting FRβ-expressing TAMs with an anti-mouse FRβ monoclonal antibody conjugated to *Pseudomonas* exotoxin A significantly depleted TAMs and reduced tumor growth [17]. A folate-conjugated TLR7 agonist against FRβ-expressing macrophages not only showed in vivo activity in a range of tumor models, including metastatic lesions, but also reversed the expression of a high M2-like to M1-like macrophage ratio and increased the infiltration of cytotoxic CD8 T cells [15].

Our recent studies in a syngeneic mouse model of high-grade serous ovarian cancer (BR-Luc [178,179]) treated with the FR-targeted pyrrolo[2,3-*d*]pyrimidine inhibitor AGF94 (Figure 2B, Table 2) [147] demonstrated anti-tumor efficacy, accompanied by a direct impact on the TME, including significantly decreased FRβ-expressing TAMs and no evidence of CD3+ T cell depletion or impact on the relative proportions of CD4+ and CD8+ T cells after drug treatment (A. Wallace-Povirk, L. Rubinsak, A. Malysa, S.H. Dzinic, M. Ravindra, C. O’Connor, Z. Hou, S. Kim, J. Back, L. Polin, et al., manuscript submitted). As AGF94 is transported by both FRs and the PCFT [147], this could represent a new approach for therapy of high-grade serous ovarian cancer through its ability to directly target the tumor via uptake by FRα and/or the PCFT and its effects on the TME, particularly FRβ-expressing TAMs.

AGF94 is an inhibitor of de novo purine biosynthesis at GARFTase [147]; this raises the intriguing question of whether C1 metabolism could represent in a unique vulnerability for TAMs, in addition to the primary tumor. Serine metabolism was associated with an inflammatory response in macrophages via its impact on glutathione synthesis [180], and depletion of S-adenosyl methionine was reported to inhibit inflammatory macrophages [181], suggesting an important role for C1 metabolism in TAM biology. Future studies will be necessary to further explore the potential of targeting C1 metabolism in inflammatory macrophages with a new generation of C1 inhibitors as a novel therapeutic strategy in EOC.

## 7. Conclusions

The review summarizes the advances in the biology and treatment of EOC with FRα- and PCFT-targeted therapeutics. Although studies involving FRα-targeted therapies with antibodies (such as farletuzumab) [111,112,113,114], antibody-drug conjugates (such as mirvetuximab soravtansine) [119], or drug conjugates (such as EC145) [5] combined with high frequency FRα expression have established FRα as a viable and tumor-specific approach for treating EOC, clinical results have been mixed [110,115,116,118,120,124,125]. This, in part, reflects the wide range of FRα expression and the challenges in predicting which patients are most likely to respond to FRα-targeted therapies.

Of particular interest are therapies targeting C1 metabolism in relation to EOC, such as CT900, which is transported by FRα and inhibits thymidylate synthase [26], as CT900 has shown promising single-agent activity in early phase clinical trials [126]. Of further interest is the potential of treating EOC with FRα and the PCFT dual-targeted antifolates, such as AGF94, that derive their tumor selectivity from their FRα and PCFT specificities over the ubiquitously expressed RFC [14,41,147,148,149]. The near constitutive expression of the PCFT in EOC [14], combined with its tumor specificity and the limited RFC transport, would result in fewer toxicities than chemotherapy, making this approach highly appealing. As an anti-purine biosynthesis inhibitor for EOC, AGF94 would inhibit cells independent of wild-type/mutant *p53* and *BRCA* status [138,139], show selectivity based on impaired purine salvage [140,141] and suppress mTOR signaling [142,143]. With the recognition of the importance of mitochondrial C1 catabolism from serine to the malignant phenotype, including ovarian cancer, an exciting new generation of investigational agents which target this pathway (e.g., SHIN2 and AGF347) is being developed [150,151,153,155], some of which are transported by FRα and the PCFT and potently inhibit EOC cells [156].

While EOC is considered an immunologically “cold” tumor, there is substantial interest in targeting the TME given its role in disease progression and therapy resistance in high-grade serous EOC [175]. Particularly exciting is the use of engineered CAR T cells containing a FRα-specific epitope linked to T cell receptor [176], and the development of novel FR-targeted cytotoxic conjugates or pyrrolopyrimidine antifolates such as AGF94 [15,16,17,18,147]. The latter could offer an entirely new approach for EOC therapy through its multi-targeting of the tumor via FRα and the PCFT and TAMs via FRβ. Of particular interest will be better understanding the metabolic basis for the relationship of C1 metabolism to inflammatory responses mediated by TAMs.

FR- and C1-targeted therapeutics, as described in this review, have extraordinary potential to improve the treatment of patients with EOC. The excitement is palpable as work progresses to demonstrate whether this potential for combatting this devastating disease can be realized.
